# Can *Alhaji maurorum* as a halophyte plant be ensiled with molasses and *Saccharomyces cerevisiae* well?

**DOI:** 10.1186/s13568-023-01529-8

**Published:** 2023-03-04

**Authors:** Mohsen Kazemi, Reza Valizadeh

**Affiliations:** 1 Department of Animal Science, Faculty of Agriculture and Animal Science, University of Torbat-e Jam, Torbat-e Jam, Iran; 2 Department of Animal Science, Faculty of Agriculture, Ferdowsi University of Mashhad, Mashhad, Iran

**Keywords:** Caspian manna, Molasses, Nutritional value, *Saccharomyces cerevisiae*, Silage

## Abstract

*Alhagi maurorum* (Caspian Manna, AM) is a species of legume found commonly in the semi-arid region of the world. Nutritional aspects of silage prepared from AM have not been scientifically investigated so far, therefore, in this study, chemical-mineral composition, gas production parameters, ruminal fermentation parameters, buffering capacity, and silage characteristics of AM were investigated by standard laboratory methods. Fresh AM was ensiled in the mini-silos (3.5 kg) and treated with (1) no additive (control), (2) 5% molasses, (3) 10% molasses, (4) 1 × 10^4^ CFU of *Saccharomyces cerevisiae* [SC]/g of fresh silage, (5) 1 × 10^4^ CFU of SC/g of fresh silage + 5% molasses, (6) 1 × 10^4^ CFU of SC/g of fresh silage + 10% molasses, (7) 1 × 10^8^ CFU of SC/g of fresh silage, (8) 1 × 10^8^ CFU of SC/g of fresh silage + 5% molasses, and (9) 1 × 10^8^ CFU of SC/g of fresh silage + 10% molasses for 60 days. The lowest concentrations of NDF and ADF were related to treatments no. 6 and 5, respectively (p < 0.0001). The ash content as well as sodium, calcium, potassium, phosphorus and magnesium were highest in treatment no 2. Silages containing 10% molasses (no. 3) had the highest and lowest lactic (p < 0.0001) and butyric (p < 0.0001) acids, respectively. The highest amount of potential gas production was observed in treatments no. 5 and 6, respectively (p < 0.0001). Total yeast was decreased with increasing molasses in the silages (p < 0.0001). Acid-base buffering capacity was also highest in treatments no. 6 and 5, respectively (p = 0.0003). In general, due to the fibrous nature of AM, it is recommended to add molasses at levels of 5 or 10% when ensiling. The silages containing SC at a lower level (1 × 10^4^ CFU) along with higher levels of molasses (10% of DM) had better ruminal digestion-fermentation characteristics compared to other silages. Also, the addition of molasses improved the internal fermentation characteristics of AM in the silo.

## Introduction

Some drought-resistant plants have a special nutritional value which supplies a cheap source of nutrition for small ruminants and can be used as an alternative source for conventional fodders (Kazemi and Ghasemi Bezdi 2021). *Alhagi maurorum* (AM, belonging to *Leguminosae*) is a highly branched spiny shrub whose its height reaches up to 1.5–4 feet (Ahmad et al. 2015). In a study, it was reported that AM collected in each of the three growth stages can easily meet the nutrient requirements of lactating ewes at the maintenance level (Kazemi and Ghasemi Bezdi 2021). Ensiling is a good method for preserving fresh forages in anaerobic conditions (Weinberg et al. 2010). Different additives have been used to increase the speed of pH reduction after ensiling, improve silage fermentation and increase the digestibility of fodders (Muck et al. 2018; Kazemi et al. 2019, 2022; Zhu et al. 2022). Based on Contreras et al. (2020) reports, the addition of SC to barley silage (5, 10, and 15 g/kg fresh matter) had a detrimental effect on the nutritional quality of barley after ensiling. Inoculation of corn silage with different strains of *Saccharomyces* did not affect the nutritional quality or aerobic stability (Duniere et al. 2015). It has been reported that some microbial additives such as *Saccharomyces cerevisiae* (SC), which are directly added to ruminant diets, improve rumen health and feed efficiency and reduce methane production (Tristant et al. 2015; Elmetwaly et al. 2022; McAllister et al. 2011). Consequently, the addition of SC to silage instead of adding it to the ration may be a convenient method for animal nutritionists to deliver these beneficial microbes into the ruminal. The SC can be defined as fourth-generation silage inoculants and it can have beneficial effects on the host animal, while other inoculants such as lactic acid bacteria can enhance silage fermentation characteristics. Molasses is an additive that is commonly added to the ensiled forages as a source of readily fermentable carbohydrates to increase the fermentation characteristics of prepared silages (Yunus et al. 2000; Lima et al. 2010). Also, it has been reported that molasses can compensate for the water-soluble carbohydrates loss caused by the initial undesirable bacteria activity and increase the substrate for lactic acid fermentation during ensiling (Zhang et al. 2022). There was no scientific data about the AM forage after ensiling with or without additives. So, the objectives of this experiment were to evaluate the effect of inoculation of AM with SC alone or in combination with molasses (5 or 10%) on chemical-mineral composition, in vitro gas production, buffering capacity, and digestion-fermentation characteristics of AM silage.

## Materials and methods

### Plant collection, silage preparation, and treatments

The whole part of AM plant was gathered from the semi-arid rangeland of Torbat-e Jam in July 2021. This area is 8184 square kilometers, from 60° 15´E to 60° 30´E, and 34° 35´N to 35° 47´N. In average, its height is 928 m above sea level. The samples were chapped with a laboratory chopper into pieces of about 2 cm. The chapped samples were ensiled in a 3.5 kg polyethylene mini-silos for 60 days. Fresh AM was ensiled in the mini-silos (3.5 kg) and treated with 1] no additive (control, AM1), 2] 5% molasses (AM2), 3] 10% molasses (AM3), 4] 1 × 10^4^ CFU (colony forming units)/g of fresh silage (AM4), 5] 1 × 10^4^ CFU of SC/g of fresh silage + 5% molasses (AM5), 6] 1 × 10^4^ CFU of SC/g of fresh silage + 10% molasses (AM6), 7] 1 × 10^8^ CFU of SC/g of fresh silage (AM7), 8] 1 × 10^8^ CFU of SC/g of fresh silage + 5% molasses (AM8), 9] 1 × 10^8^ CFU of SC/g of fresh silage + 10% molasses (AM9) for 60 days. Five replicates were considered for each treatment. Kimiazyme Company (Tehran, Iran) prepared the SC (8 × 10^10^ CFU/g of fresh material) with the trade name Zy-MOS Ultra.

### Silage sampling, silage fermentation, and chemical-mineral analysis

After 60 days of ensiling, the silos were opened. Sampling was done from each silo separately. For dry matter (DM) determination, a fresh sample of each silage was dried in an air-forced oven at 105 °C for 24 h. The pH of the silage extract was determined by a pH meter (Hana, Model HI 2210-01, USA) according to Eyni and Bashtani (2016). The amount of 10 ml of silage extract was mixed with 10 ml of 0.2 N HCl and preserved in the freezer at −18 °C for ammonia nitrogen analysis. The concentrations of lactic, acetic, propionic, and butyric acids and ethanol in silage extract were determined by a KNAUER HPLC system equipped with a UV-VIS detector (Azura, Germany) and with a C18 column (25 cm × 4.6 mm id, 5 μm). The operation was run by a mobile phase of 0.005 mM H_2_SO_4_ with a flow rate of 0.5 mL/min. The phosphorus content was determined by a spectrophotometer (UV-Vis array Spectrophotometer, Photonix-Ar-2017, Iran) using the molybdovanadate method. The mineral concentrations of silage samples, including calcium, sodium, potassium, magnesium, manganese, iron, and zinc were measured by atomic absorption spectrometry (SavantAA, GBC, Australia). A Soxhlet extracting apparatus (AOAC 2005) was employed for ether extract (EE) determination. The concentrations of acid detergent fiber (ADF) and neutral detergent fiber (NDF) were measured according to the procedure of Ankom technology (Ankom Technology 2006a, b) using the solutions recommended by Van Soest et al. (1991). The crude protein (CP) content of samples was determined according to the Kjeldahl method (AOAC 2005). The concentration of non-fiber carbohydrates (NFC) was determined by subtracting CP, NDF, EE, and ash from total DM (Sniffen et al. 1992). By using an electric furnace, the ash content of samples was determined at the temperature of 550 °C for 4 h. The preparation of samples and enumeration of yeast was done according to Duniere et al. (2015).

### Laboratory and in vitro methods

The rumen fluid was gathered from two ruminal fistulated lambs that were fed by corn silage, wheat straw, and by a commercial concentrate twice (6:30 am and 17:30) a day at the maintenance level. The collected rumen fluid was filtered via four layers of cheesecloth and then immediately transferred to the central laboratory for further analysis. The gas test was run according to Menke and Steingass (1988) protocol in two different runs. The amount of 200 mg of each silage sample was transferred into the 100 ml glass syringes. The instruction of Menke and Steingass (1988) was employed for artificial saliva preparation. With a ratio of 1:2 (rumen fluid to artificial saliva solution), each glass syringe was filled. The tube connected to the syringe outlet was plumped by a plastic clip. Each syringe was then gently shaken and placed in a water bath at 39 °C for 3, 6, 9, 12, 24, 48, 72, and 96 h of incubation (Menke and Steingass 1988; Kazemi et al. 2021). A medium similar to the gas test was used to determine pH and ammonia nitrogen concentrations after 24 h incubation. The method of Jasaitis et al. (1987) was employed for buffering capacity parameters determination. In brief, 0.5 g DM of each sample was weighted into a beaker, added 50 ml distilled deionized water and then stirred continuously with a magnetic stir bar. Buffering capacity was determined by addition of acid (0.1 N HC1) or base (0.1 N NaOH) until the pH was decreased to 4 or increased to 9, respectively. Initial pH and all further measurements were recorded when the solution reached the equilibration point after 3 min. A gas test-like culture medium was used to determine total volatile fatty acids (TVFA), pH, and ammonia nitrogen concentrations after 24 h incubation. The procedure of sampling for TVFA determination was conducted according to the protocol of Getachew et al. (2004). The method suggested by Barnett and Reid (1957) with the use of the Markham device (1942) was employed for TVFA determination. The 24 h in vitro dry matter digestibility (IVDMD) and organic matter digestibility (IVOMD) of silage samples was determined according to Kazemi and Ghasemi Bezdi (2021) protocol. The method described by Komolong et al. (2001) was used for ammonia nitrogen analysis.

### Statistical analysis and equations

The equations described by Menke and Steingass (1988) were employed for the estimation of metabolizable energy (ME) and net energy for lactation (NEl) via 24 h gas production, CP, and EE. All parameters were replicated 5 times. All data were analyzed in a completely randomized design using the GLM procedure of SAS (2002). The statistical differences between means were calculated by Duncan’s multiple range test (Kazemi et al. 2012). The fractional rate of gas production (c_gas_, %/h) and potential gas production (b_gas_, ml/h) were determined by a nonlinear equation [Ørskov and McDonald 1979; $Y=b\left(1-{e}^{-ct}\right)$], in which Y is the volume of gas produced at time t.

## Results

### Chemical and mineral composition

The Chemical compositions of different silages prepared from AM are shown in Table 1. The contents of DM and EE were not affected by the treatments (p > 0.05), however, the concentrations of NDF, ADF, CP, Ash, and NFC were affected by the treatments (p < 0.0001). The silage containing 1 × 10^4^ CFU of SC + 10% molasses (AM6) had the highest CP (12.1% of DM) and NFC (22.9% of DM), and lowest NDF (55.5% of DM) and ADF (46.9% of DM) compared to the control silage (p < 0.0001).

**Table 1:** Chemical compositions (% of DM) of different silages prepared from *Alhaji maurorum*

Item	DM	CP	NDF	ADF	EE	Ash	NFC
AM1	34.2	11.7^ab^	64.2^ab^	59.6^a^	1.19	10.6^b^	12.3^f^
AM2	34.2	11.7^ab^	64.8^a^	55.4^bc^	1.24	11.1^a^	10.1^g^
AM3	35.3	11.4^bc^	60.4^de^	53.3^cd^	1.26	9.16^cd^	17.8^cd^
AM4	35.5	10.9^def^	59.5^de^	52.9^d^	1.24	9.38^c^	19.0^bc^
AM5	36.0	10.5^f^	58.7^e^	51.9^d^	1.25	8.82^d^	20.8^b^
AM6	36.3	12.1^a^	55.5^f^	46.9^e^	1.27	8.31^e^	22.9^a^
AM7	34.2	11.4^bcd^	61.1^cd^	53.4^cd^	1.26	10.2^b^	16.0^de^
AM8	35.3	11.0^cde^	62.6^bc^	54.3^bcd^	1.28	9.49^c^	15.6^e^
AM9	34.2	10.8^ef^	63.7^b^	56.3^b^	1.27	9.21^cd^	15.0^e^
SEM	0.42	0.095	0.54	0.59	0.02	0.14	0.66
P-value	0.92	< 0.0001	< 0.0001	< 0.0001	0.71	< 0.0001	< 0.0001

^a-g^Means within the same columns followed by different letters differ significantly at the P-value indicated

*AM1 Alhaji maurorum* after ensiling (no additive, control), *AM2 Alhaji maurorum* ensiled with 5% molasses, *AM3 Alhaji maurorum* ensiled with 10% molasses, *AM4 Alhaji maurorum* ensiled with 1 × 10^4^ CFU of *Saccharomyces cerevisiae*, *AM5 Alhaji maurorum* ensiled with 1 × 10^4^ CFU of *Saccharomyces cerevisiae* +  + 5% molasses, *AM6 Alhaji maurorum* ensiled with 1 × 10^4^ CFU of *Saccharomyces cerevisiae* + 10% molasses, *AM7 Alhaji maurorum* ensiled with 1 × 10^8^ CFU of *Saccharomyces cerevisiae*, *AM8 Alhaji maurorum* ensiled with 1 × 10^8^ CFU of *Saccharomyces cerevisiae* + 5% molasses, *AM9 Alhaji maurorum* ensiled with 1 × 10^8^ CFU of *Saccharomyces cerevisiae* + 10% molasses, *DM (% of fresh weight)* dry matter, *CP* crude protein, *NDF* neutral detergent fiber, *ADF* acid detergent fiber, *EE* ether extract, *NFC* non-fiber carbohydrate, *SEM* standard error of the mean

The mineral compositions of different silages prepared from AM are presented in Table 2. All measured minerals were affected by the treatments (p < 0.05). Macro elements (such as sodium, calcium, phosphorus, potassium, and magnesium) were highest in silages containing only 5% molasses (p < 0.05), however trace minerals including manganese, iron, and zinc were highest in silages containing 1 × 10^8^ CFU of SC/g of fresh forage (p < 0.05).

**Table 2:** Mineral compositions of different silages prepared from *Alhaji maurorum*

Item	Na	K	Ca	P	Mg	Mn	Zn	Fe
AM1	1.61^bc^	11.5^a^	13.2^abc^	4.39^abc^	5.45^a^	36.2^ab^	18.7^ab^	359^bc^
AM2	2.02^a^	11.6^a^	13.8^a^	4.62^a^	5.56^a^	33.3^bc^	18.6^ab^	353^bc^
AM3	1.80^ab^	9.73^b^	11.1^d^	3.68^d^	4.30^bc^	25.4^e^	15.7^b^	238^d^
AM4	1.31^de^	9.33^bc^	12.0^abcd^	3.99^abcd^	4.64^b^	32.4^bcd^	17.4^ab^	367^bc^
AM5	1.28^e^	7.82^d^	11.1^cd^	3.72^cd^	3.89^c^	28.9^de^	15.3^b^	327^bc^
AM6	1.69^b^	8.17^cd^	11.7^bcd^	3.89^bcd^	3.84^c^	29.2^cde^	15.7^b^	314^c^
AM7	1.39^cde^	9.89^b^	13.1^abcd^	4.38^abc^	4.36^bc^	38.2^a^	19.2^a^	488^a^
AM8	1.56^bcd^	9.26^bc^	13.4^ab^	4.48^ab^	4.01^c^	34.1^ab^	18.3^ab^	406^b^
AM9	1.73^b^	8.91^bcd^	12.4^abcd^	4.15^abcd^	3.93^c^	32.1^bcd^	17.5^ab^	369^bc^
SEM	0.051	0.27	0.25	0.08	0.13	0.81	0.39	14.13
P-value	0.0002	< 0.0001	0.04	0.03	< 0.0001	< 0.0001	0.05	< 0.0001

^a-e^Means within the same columns followed by different letters differ significantly at the P-value indicated

*AM1 Alhaji maurorum* after ensiling (no additive, control), *AM2 Alhaji maurorum* ensiled with 5% molasses, *AM3 Alhaji maurorum* ensiled with 10% molasses, *AM4 Alhaji maurorum* ensiled with 1 × 10^4^ CFU of *Saccharomyces cerevisiae*, *AM5 Alhaji maurorum* ensiled with 1 × 10^4^ CFU of *Saccharomyces cerevisiae* +  + 5% molasses, *AM6 Alhaji maurorum* ensiled with 1 × 10^4^ CFU of *Saccharomyces cerevisiae* + 10% molasses, *AM7 Alhaji maurorum* ensiled with 1 × 10^8^ CFU of *Saccharomyces cerevisiae*, *AM8 Alhaji maurorum* ensiled with 1 × 10^8^ CFU of *Saccharomyces cerevisiae* + 5% molasses, *AM9 Alhaji maurorum* ensiled with 1 × 10^8^ CFU of *Saccharomyces cerevisiae* + 10% molasses, *Na (g/kg DM)* sodium, *K (g/kg DM)* potassium, *Ca (g/kg DM)* calcium, *P (g/kg DM)* phosphorus, *Mg (g/kg DM)* magnesium (g/kg DM), *Mn (mg/kg DM)* manganese, *Zn (mg/kg DM)* zinc, *Fe (mg/kg DM)* iron, *SEM* standard error of the mean

### Fermentation characteristics of prepared silages

The fermentation characteristics of different silages prepared from AM are exhibited in Table 3. Different fermentation characteristics were observed among the prepared silages (p < 0.05). The silages containing only 10% molasses (AM3) had the lowest total yeast (p < 0.0001), acid acetic (p = 0.006), ethanol (p < 0.0001), pH (p = 0.002), and highest lactic and propionic acids (p < 0.0001). The ammonia nitrogen in the silage was not affected by the treatments (p > 0.05).

**Table 3:** Fermentation characteristics of different silages prepared from *Alhaji maurorum*

Item	TY	LA	AA	PA	BA	AN	E	pH
AM1	4.10^e^	0.45^ed^	0.17^abcd^	0.027^cd^	0.072^ cd^	0.185	0.152^cd^	5.47^a^
AM2	3.60^f^	0.60^cb^	0.14^bcd^	0.067^ab^	0.053^de^	0.175	0.130^d^	5.40^abc^
AM3	3.20^g^	0.72^a^	0.12^d^	0.082^a^	0.032^e^	0.162	0.112^d^	5.32^d^
AM4	4.81^d^	0.39^e^	0.17^abc^	0.020^cd^	0.110^b^	0.192	0.242^b^	5.42^ab^
AM5	4.29^e^	0.52^ cd^	0.15^bcd^	0.045^bc^	0.048^de^	0.185	0.192^c^	5.36^bcd^
AM6	4.19^e^	0.66^ab^	0.13^ cd^	0.020^cd^	0.041^de^	0.165	0.155^cd^	5.34^cd^
AM7	8.83^a^	0.37^e^	0.20^a^	0.011^d^	0.160^a^	0.212	0.350^a^	5.47^a^
AM8	8.31^b^	0.49^d^	0.18^ab^	0.036^cd^	0.102^bc^	0.175	0.285^b^	5.42^ab^
AM9	7.80^c^	0.59^cb^	0.16^abcd^	0.027^cd^	0.075^ cd^	0.192	0.242^b^	5.38^bcd^
SEM	0.35	0.02	0.02	0.004	0.007	0.005	0.01	0.012
P-value	< 0.0001	< 0.0001	0.006	< 0.0001	< 0.0001	0.46	< 0.0001	0.002

^a-g^ Means within the same columns followed by different letters differ significantly at the P-value indicated

*AM1 Alhaji maurorum* after ensiling ( no additive, control), *AM2 Alhaji maurorum* ensiled with 5% molasses, *AM3 Alhaji maurorum* ensiled with 10% molasses, *AM4 Alhaji maurorum* ensiled with 1 × 10^4^ CFU of *Saccharomyces cerevisiae*, *AM5 Alhaji maurorum* ensiled with 1 × 10^4^ CFU of *Saccharomyces cerevisiae* +  + 5% molasses, *AM6 Alhaji maurorum* ensiled with 1 × 10^4^ CFU of *Saccharomyces cerevisiae* + 10% molasses, *AM7 Alhaji maurorum* ensiled with 1 × 10^8^ CFU of *Saccharomyces cerevisiae*, *AM8 Alhaji maurorum* ensiled with 1 × 10^8^ CFU of *Saccharomyces cerevisiae* + 5% molasses, *AM9 Alhaji maurorum* ensiled with 1 × 10^8^ CFU of *Saccharomyces cerevisiae* + 10% molasses, *TY* total yeast (log_10_ CFU/g DM), *LA (% of DM)* lactic acid, *AA (% of DM)* acetic acid, *PA (% of DM)* propionic acid, *BA (% of DM)* butyric acid, *AN (% of total nitrogen)* ammonia nitrogen, *E (% of DM)* ethanol, *SEM* standard error of the mean

### In vitro gas test parameters and digestion-fermentation parameters obtained from the culture medium

The in vitro gas test parameters related to different silages prepared from AM are shown in Table 4. The highest amount of b_gas_ (24.3 ml/200 mg DM, p < 0.0001), 12 (p = 0.015), 24 (p = 0.002), 48 (p = 0.002), and 72 h (p < 0.0001) gas produced (3.47, 11.9, 16, and 17.6 ml/200 mg DM, respectively) were observed in silages containing 1 × 10^4^ CFU of SC/g of fresh silage + 10% molasses (AM6). The c_gas_ was not affected by the different silages (p > 0.05). Some fermentation parameters of the culture medium following the incubation of different silage prepared from AM are exhibited in Table 5. The ammonia nitrogen of the culture medium wasn’t different among the different silage prepared from AM (p > 0.05). The highest concentrations of TVFA (69.2 mmol/L, p = 0.05), IVDMD (43.4%, p = 0.0008), IVOMD (47.7, p = 0.002), ME (4.55 MJ/Kg DM, p = 0.003), and NEl (2.16 MJ/Kg DM, p = 0.003), as well as the lowest amount of 24 h pH (6.37, p = 0.002) was related to the silages containing 1 × 10^4^ CFU of SC/g of fresh silage + 10% molasses (AM6).

**Table 4:** The in vitro gas test parameters related to different silages prepared from *Alhaji maurorum*

Item	b_gas_	c_gas_	Gas 12 h	Gas 24 h	Gas 48 h	Gas 72 h
AM1	8.99^e^	0.0088	0.067^c^	2.67^d^	2.67^d^	2.68^f^
AM2	9.33^e^	0.0084	0.133^bc^	2.50^d^	3.0^d^	3.64^ef^
AM3	14.9^cd^	0.0172	1.63^abc^	6.57^bcd^	8.93^bcd^	9.27^cd^
AM4	19.0^b^	0.0188	1.93^abc^	9.77^abc^	12.2^abc^	13.3^abc^
AM5	22.9^a^	0.0174	1.84^abc^	10.8^ab^	14.2^ab^	15.5^ab^
AM6	24.3^a^	0.0206	3.47^a^	11.9^a^	16.0^a^	17.6^a^
AM7	13.5^d^	0.0130	1.50^bc^	4.57^d^	6.90^cd^	7.73^de^
AM8	18.1^bc^	0.0191	2.0^ab^	7.03^abcd^	11.4^abc^	12^bcd^
AM9	15.4^bcd^	0.0150	1.10^bc^	4.94^cd^	9.27^abcd^	9.84^cd^
SEM	1.05	0.0016	0.25	0.76	1.03	1.03
P-value	< 0.0001	0.59	0.015	0.002	0.002	< 0.0001

^a-^^f^ Means within the same columns followed by different letters differ significantly at the P-value indicated

*AM1 Alhaji maurorum* after ensiling (no additive, control), *AM2 Alhaji maurorum* ensiled with 5% molasses, *AM3*:*Alhaji maurorum* ensiled with 10% molasses, *AM4 Alhaji maurorum* ensiled with 1 × 10^4^ CFU of *Saccharomyces cerevisiae*, *AM5 Alhaji maurorum* ensiled with 1 × 10^4^ CFU of *Saccharomyces cerevisiae* +  + 5% molasses, *AM6 Alhaji maurorum* ensiled with 1 × 10^4^ CFU of *Saccharomyces cerevisiae* + 10% molasses, *AM7 Alhaji maurorum* ensiled with 1 × 10^8^ CFU of *Saccharomyces cerevisiae*, *AM8 Alhaji maurorum* ensiled with 1 × 10^8^ CFU of *Saccharomyces cerevisiae* + 5% molasses, *AM9 Alhaji maurorum* ensiled with 1 × 10^8^ CFU of *Saccharomyces cerevisiae* + 10% molasses, *b*_*gas*_* (ml/200 mg DM)* potential gas production, *c*_*gas*_* (%/h)* fractional rate of gas production, *gas 12, 24, 48, and 72 h (ml/200 mg DM)* cumulative gas production after 12, 24, 48, and 72 h incubation, *SEM* standard error of the mean

**Table 5:** Some fermentation parameters of the culture medium following the incubation of different silage prepared from *Alhaji maurorum*

Item	TVFA	NH_3_-N	pH 24 h	IVOMD	ME	NEl	IVDMD
AM1	65.3^b^	17.1	6.55^ab^	34.1^c^	3.26^d^	1.25^d^	30.4^d^
AM2	66.5^b^	17.0	6.52^ab^	33.5^c^	3.25^d^	1.25^d^	29.5^d^
AM3	67.5^ab^	16.8	6.55^ab^	40.0^b^	3.79^bcd^	1.63^bcd^	36.0^bc^
AM4	67.1^ab^	16.3	6.49^ab^	45.7^a^	4.19^abc^	1.92^abc^	40.7^ab^
AM5	67.5^ab^	16.1	6.45^bc^	46.2^a^	4.31^ab^	2.0^ab^	41.0^ab^
AM6	69.2^a^	16.1	6.37^c^	47.7^a^	4.55^a^	2.16^a^	43.4^a^
AM7	65.9^b^	16.4	6.59^a^	35.5^bc^	3.52^cd^	1.44^cd^	31.7^dc^
AM8	66.3^b^	16.2	6.53^ab^	37.0^bc^	3.83^bcd^	1.66^bcd^	33.2^dc^
AM9	66.7^b^	16.4	6.56^a^	35.2^bc^	3.54^cd^	1.45^cd^	32.0^dc^
SEM	0.28	0.30	0.014	1.34	0.10	0.07	1.24
P-value	0.05	0.12	0.002	0.002	0.003	0.003	0.0008

^a-d^Means within the same columns followed by different letters differ significantly at the P-value indicated

OMD and DMD were determined after 24 h incubation

*AM1 Alhaji maurorum* after ensiling (no additive, control), *AM2 Alhaji maurorum* ensiled with 5% molasses, *AM3 Alhaji maurorum* ensiled with 10% molasses, *AM4 Alhaji maurorum* ensiled with 1 × 10^4^ CFU of *Saccharomyces cerevisiae*, *AM5 Alhaji maurorum* ensiled with 1 × 10^4^ CFU of *Saccharomyces cerevisiae* +  + 5% molasses, *AM6 Alhaji maurorum* ensiled with 1 × 10^4^ CFU of *Saccharomyces cerevisiae* + 10% molasses, *AM7 Alhaji maurorum* ensiled with 1 × 10^8^ CFU of *Saccharomyces cerevisiae*, *AM8 Alhaji maurorum* ensiled with 1 × 10^8^ CFU of *Saccharomyces cerevisiae* + 5% molasses, *AM9 Alhaji maurorum* ensiled with 1 × 10^8^ CFU of *Saccharomyces cerevisiae* + 10% molasses, *NH*_*3*_*-N (mg/dL)* ammonia nitrogen, *TVFA (mmol/L)* total volatile fatty acid, *IVOMD (%) * in vitro organic matter digestibility after 24 h incubation, *ME (MJ/kg DM)* metabolizable energy, *NEl (MJ/kg DM)* net energy for lactation, *IVDMD (%) * in vitro dry matter digestibility after 24 h incubation, *SEM* standard error of the mean

### The pH of the extract and buffering capacity

The pH of the extract and buffering capacity parameters of different silage prepared from AM are shown in Table 6. The lowest amount of pH extract (5.64, p < 0.0001) and highest contents of acid-buffering capacity (66 mE×10^− 3^/kg DM, p < 0.0001) and titratable alkalinity (224 mE×10^− 3^/kg DM, p < 0.0001) was related to the silages containing 1 × 10^4^ CFU of SC/g of fresh silage + 10% molasses (AM6, p < 0.0001). The lowest amount of base buffering capacity (35.7 mE×10^− 3^/kg DM, p < 0.0001) and acid-base buffering capacity (76.8 mE×10^− 3^/kg DM, p = 0.0003) were related to the control silage and silages containing 1 × 10^8^ CFU of SC + 10% molasses, respectively.

**Table 6:** The pH of the extract and buffering capacity (mEq × 10^–3^) parameters of different silage prepared from *Alhaji maurorum*

Item	pH	Titratable acidity	Acid-buffering capacity	Titratable alkalinity	Base-buffering capacity	Acid–base buffering capacity
AM1	6.57^a^	138^a^	53.9^b^	80.3^ g^	35.7^f^	90.9^ cd^
AM2	6.63^a^	147^a^	54.9^b^	95.3^f^	43.4^e^	101^cb^
AM3	6.98^f^	126^b^	63.7^a^	150^ed^	52.0^d^	120^ab^
AM4	6.14^e^	121^bc^	56.8^b^	159^ cd^	59.9^b^	118^ab^
AM5	5.92^f^	112^dc^	58.2^b^	203^b^	70.8^a^	130^a^
AM6	5.64^ g^	108^d^	66.0^a^	224^a^	69.3^a^	131^a^
AM7	6.48^b^	139^a^	55.8^b^	143^e^	53.4^dc^	110^abc^
AM8	6.24^d^	105^d^	46.7^c^	165^c^	57.8^bc^	102^cb^
AM9	6.35^c^	94.3^e^	40.1^d^	143^e^	54.3^dc^	76.8^d^
SEM	0.06	3.35	1.52	8.51	2.11	3.82
P-value	< 0.0001	< 0.0001	< 0.0001	< 0.0001	< 0.0001	0.0003

^a-^^g^ Means within the same columns followed by different letters differ significantly at the P-value indicated

*AM1 Alhaji maurorum* after ensiling (no additive, control), *AM2 Alhaji maurorum* ensiled with 5% molasses, *AM3 Alhaji maurorum* ensiled with 10% molasses, *AM4 Alhaji maurorum* ensiled with 1 × 10^4^ CFU of *Saccharomyces cerevisiae*, *AM5 Alhaji maurorum* ensiled with 1 × 10^4^ CFU of *Saccharomyces cerevisiae* +  + 5% molasses, *AM6 Alhaji maurorum* ensiled with 1 × 10^4^ CFU of *Saccharomyces cerevisiae* + 10% molasses, *AM7 Alhaji maurorum* ensiled with 1 × 10^8^ CFU of *Saccharomyces cerevisiae*, *AM8 Alhaji maurorum* ensiled with 1 × 10^8^ CFU of *Saccharomyces cerevisiae* + 5% molasses, *AM9 Alhaji maurorum* ensiled with 1 × 10^8^ CFU of *Saccharomyces cerevisiae* + 10% molasses, *SEM* standard error of the mean

## Discussion

### Chemical and mineral composition

To our knowledge, the nutritional characteristics of silage prepared from AM have not been investigated so far, therefore the present data can be helpful for animal nutritionists. Different inoculants have been used to improve the quality of different silages (Dong et al. 2020; Irawan et al. 2021; Wang et al. 2022). Despite the use of different bacterial inoculations in different silage, live yeast especially SC has been less used for the improvement of silage quality. Except for NDF and ADF, other chemical compositions of the AM silage (DM, EE, and Ash) used in the present experiment were similar to values reported by Kazemi and Ghasemi Bezdi (2021). The DM range of the AM silages was between 34.2 and 36.3%, which was within an optimal range reported by Ergün et al. (2001) in different silages. In the present study, the silages containing SC as a fourth-generation inoculant at a low level (1 × 10^4^ CFU) with 10% molasses had the highest content of CP among the other silages. However, it has been reported that SC has a high ability to ferment sugars (Oude Elferink et al. 2001). Inoculation of corn silage with different strains of SC (1 × 10^3 ^CFU/g fresh weight) did not affect the nutritional quality or aerobic stability (Duniere et al. 2015). These strains also should not promote the growth of undesirable microorganisms such as molds (Duniere et al. 2015). So, the increase in the CP content of AM6 can be attributed mainly to the effects of molasses addition. In contrast with the report of Duniere et al. (2015), we observed a decrease in NDF, ADF, and Ash and an increase in NFC contents of silages following inoculation with SC at a low level (1 × 10^4^ CFU). Both NDF and ADF contents in 10% molasses silages (AM3 and AM6) were significantly lower than the control silage (p < 0.0001). The decreases in NDF and ADF content in molasses silage may be due to the effect of molasses on promoting silage fermentation (Mcdonald et al. 1991; Baytok et al. 2005). It also appears that a reduction in the NDF content of 10% molasses-treated silages was due to partial acid hydrolysis of hemicellulose (Muck and Kung 1997). After 60 d of ensiling, the AM6 silage had a lower ash concentration than AM1 and other silages. This might be explained by the significantly higher level of NFC observed in this silage.

Considering that the highest amount of raw ash was related to AM ensiled only with 5% molasses (AM2), accordingly, the concentrations of macro elements in this treatment (such as sodium, potassium, calcium, and magnesium) were also higher than in other treatments. Consuming more non-fibrous carbohydrates in AM2 silage can be the main reason for increasing the amount of ash followed by micro-elements in this treatment.

### Fermentation characteristics of prepared silages

In line with the report of Zhao et al. (2019), we found an increase in lactic and propionic acid concentrations and a decrease in butyric and acetic acids in silages containing molasses. A high level of ethanol was detected in the silages ensiled with high levels of SC (1 × 10^8^ CFU of SC/g of fresh silage) and this could be explained by the relatively high DM of AM and activity of yeasts. Also, it has been reported that ethanol is commonly produced in silage with high DM (Hengeveld 1983). In line with the report of Zhao et al. (2019), Moreover, less ethanol in AM1 and AM2 compared to SC inoculation silages may be related to an inhibition of yeasts activities by lactic acid-producing bacteria. Consistence with the report of Zhao et al. (2019), the higher lactic acid in silages containing only 10% molasses (AM3) could be attributed to inhibition of undesired microbial growth and acid hydrolysis of available structural carbohydrates, which were reflected in the low pH.

### In vitro gas test parameters and fermentation parameters obtained from the culture medium

The in vitro gas test as an easily and low-cost method is commonly applied as an indicator of the efficiency of ruminal digestibility and predicts the ME of animal feed (Contreras-Govea et al. 2011; Kazemi et al. 2022). In this study, gas 12, 24, 48, and 72 h and also the b_gas_ of some prepared silages was increased compared to the control and AM2 silages, indicating that in vitro ruminal gas production can be increased by the addition of molasses or SC inoculation. Moreover, SC addition (in the level of 1 × 10^4^ CFU of SC/g of fresh silage) rather than molasses (in 5 or 10%) further increased the b_gas_ of resulting silages. Since the c_gas_ did not differ among the present silages, therefore, it seems that all these silages have produced gas at a relatively similar rate in the culture medium.

Similar to the reports of Zhao et al. (2019) and Moselhy et al. (2015), we found an increase in IVDMD and IVOMD following the 10% molasses addition (AM3). Ammonia nitrogen is related to the degradation of CP and amino acids, which has been taken as an indicator of the extent of proteolysis in silage. Ammonia nitrogen is related to the digestibility of CP and amino acids and is considered as indicator of the rate of protein degradation in silage. However, in this study, we observed no significant difference in ammonia nitrogen among the prepared silages. Also, the addition of SC at a low level (AM4) caused a 33 and 34% increase in the IVDMD and IVOMD, respectively compared to the control treatment. Since the ME and NEl of the studied silages have been estimated based on the 24 h gas production, CP and EE, therefore, the highest amount of ME and NEl was related to AM6. It is reported that volatile fatty acids are the main products of rumen fermentation, and they contribute approximately 70 to 80% of energy requirements (Dijkstra et al. 2005; Bergman 1990). So, we observed a higher increase of TVFA in AM6 compared to the control group which supplies more energy for ruminants. Volatile fatty acids (VFAs) and lactic acid produce in the rumen and can reduce ruminal pH. Lower pH in AM6 can be attributed to higher production of VFAs in the culture medium.

### The pH of the extract and buffering capacity

The buffering capacity of animal feed is an important physico-chemical characteristic is related to the ability of the feed ingredients to be acidified or alkalinized. The buffering capacity refers to the ability of a certain amount of feed to resist pH changes after being added to an acidic or alkaline solution (Giger-Reverdin et al. 2002). We observed a high content of acid-base buffering capacity in AM6 and AM5 which can be useful for pH balance in the rumen. If the buffering capacity of feed ingredients in the ration is low, the importance of using buffers in the feed will be highlighted. Also, knowing the buffering capacity of the feed can be effective in the decision of animal nutritionists to add buffers to the diet or not. The buffering capacity of some protein feeds and leguminous forages have been reported to be higher than 85 mEq×10^− 3^ (Montanez-Valdez et al. 2013), which is consistent with the present study for AM silages. It is reported that the amount and composition of minerals in the ash have a particular buffering effect on the plant’s initial pH (Levic et al. 2005). As a result of different ash content in the prepared silages (8.31–11.1%), their buffering capacity was also different. It is reported that the initial pH and titratable acidity are the most critical determinants of ruminal pH. In the present study, the highest titratable acidity was observed for silages treated with only 5% molasses (147 mEq×10^− 3^), indicating high resistance to acidification. Exception for AM6, other silages had a pH near the neutral zone, and therefore, their consumption couldn’t lead to rumen pH reduction.

In conclusion, additives are necessary to avoid spoilage and enhance the fermentation characteristics of fresh AM silage. The 10% molasses along with SC at low level (1 × 10^4^ CFU of SC/g of fresh silage) was more effective than higher level of SC (1 × 10^8^ CFU of SC/g of fresh silage) to improve the silage quality of AM. Molasses addition increased in vitro potential gas production of AM silage. The application of both molasses (10%) and SC (low level: 1 × 10^4^ CFU of SC/g of fresh silage) is recommended to enhance the fermentation quality, nutritive characteristics and in vitro digestibility of AM silages. The AM can be well ensiled with molasses and SC and it can provide a continuous roughage source for ruminant livestock in areas with semi-arid climate.

## Data Availability

The data will be made available upon reasonable request.

## Abbreviations

AM: *Alhagi maurorum*
SC: *Saccharomyces cerevisiae*
CFU: Colony forming units
DM: Dry matter
EE: Ether extract
ADF: Acid detergent fiber
NDF: Neutral detergent fiber
CP: Crude protein
NFC: Non fiber carbohydrates
TVFA: Total volatile fatty acids
IVDMD: In vitro dry matter digestibility
IVOMD: In vitro organic matter digestibility
ME: Metabolizable energy
NEl: Net energy for lactation
b_gas_: Potential gas production
c_gas_: Fractional rate of gas production
VFAs: Volatile fatty acids
